# Prevalence and determinants of chronic non-communicable diseases among prison inmates in the city of Tete, Mozambique: a cross-sectional study

**DOI:** 10.1186/s12889-025-24387-4

**Published:** 2025-09-24

**Authors:** Sancho Pedro Xavier, Nelson Jossefe Conde Motivo, Ramim Xavi, Nelson Domingos Cote, Ageo Mario Cândido da Silva, Audêncio Victor

**Affiliations:** 1 Institute of Collective Health, Federal University of Mato Grosso, Av. Fernando Correa da Costa, nº 2367 - Bairro Boa Esperança, Cuiabá, MT 78060-900 Brazil; 2 Faculty of Health Sciences, Zambeze University, Provincial Hospital Campus, Tete, Mozambique; 3 School of Public Health, University of São Paulo (USP), Avenida Doutor Arnaldo, 715, São Paulo, São Paulo 01246904 Brazil; 4 Department of Infectious Disease Epidemiology and International Health, London School of Hygiene & Tropical Medicine, Keppel Street, London, WC1E 7HT UK

**Keywords:** Non-communicable chronic diseases, Prevalence, Prison, Prisoners health, Mozambique

## Abstract

**Background:**

High health inequalities and non-communicable chronic diseases (NCDs) are prevalent in the prison population, particularly in Sub-Saharan African countries, posing significant implications for morbidity and mortality. This study analyzes the prevalence and determinants of these diseases among prisoners in the city of Tete, Mozambique.

**Methods:**

A cross-sectional study was conducted among inmates at the Tete City Prison, Mozambique. Diabetes Mellitus (DM) was diagnosed based on the criteria established by the International Expert Committee, which defines diabetes as a fasting plasma glucose (FPG) level of ≥ 126 mg/dL (7.0 mmol/L). Participants who reported a previous diagnosis of diabetes by a doctor or health professional were also classified as diabetic. Multivariable logistic regression was used to identify significant risk factors, reporting adjusted Odds Ratios (AOR) with a 95% confidence interval, considering a p-value < 0.05 as significant.

**Results:**

The prevalence of hypertension, DM, and obesity was 36.90% (95% CI: 31.33; 42.73), 10.34% (95% CI: 7.09; 14.44), and 1.38% (95% CI: 0.38; 3.49), respectively. A family history of DM (AOR: 14.95; 95% CI: 3.32; 67.44) and being female (AOR: 2.54; 95% CI: 1.43; 4.52) were identified as risk factors associated with DM and Hypertension, respectively.

**Conclusion:**

The study revealed high rates of DM and hypertension among inmates, highlighting family history as a risk factor for DM and the association of female with hypertension. Urgent implementation of preventive measures for hypertension, along with early screenings for individuals with a family history of DM, is essential to mitigate complications in prison environment.

**Supplementary information:**

The online version contains supplementary material available at 10.1186/s12889-025-24387-4.

## Introduction

Non-communicable chronic Diseases (NCDs) pose a significant global challenge. In the context of Sub-Saharan Africa, they exert additional strain on the already burdened health systems of the region [1]. NCDs, including hypertension, heart disease, diabetes, and obesity, play a pivotal role, contributing to elevated rates of mortality and morbidity. Notably, these conditions are more prevalent in younger population groups compared to more developed regions [1–3], with prevalence rates of 31.5% for hypertension [4], 7.3% for diabetes mellitus (DM), and 19.5% for obesity [4].

The global prison population approaches 11 million [5], highlighting the urgent need to address the human rights of inmates, as established by the UN Minimum Rules, which advocate improvements in prison conditions [6, 7]. Health disparities persist within this group, often consisting of individuals who are more marginalized and economically disadvantaged in society [8]. The prison environment amplifies health challenges, making it crucial to address not only criminal justice issues but also to implement health policies to ensure human rights and dignified living conditions during incarceration [9]. Although it is acknowledged that incarcerated individuals in this region often suffer from chronic health conditions and encounter significant barriers to accessing appropriate healthcare services [10], most health monitoring efforts have concentrated on infectious diseases, such as HIV, tuberculosis, and COVID-19,, and prison health remains neglected largely due to political sensitivities, prioritization of security, and structural barriers to healthcare access [11, 12].

Although there is a considerable amount of research on NCDs in Sub-Saharan Africa, there remains a significant gap in understanding the impact of these conditions on vulnerable populations, particularly prisoners. Studies conducted so far reveal an alarming prevalence of DM (9.4%, potentially reaching 14%), hypertension (39.6%), and obesity (9.8%) [13, 14]. To the best of our knowledge, no studies have been conducted on the incidence or prevalence of NCDs in the prison population in Mozambique. Recognizing this substantial gap in scientific knowledge regarding the prevalence of NCDs in this vulnerable group, we undertook this research to analyze the prevalence and determinants of these diseases among prisoners in the city of Tete, Mozambique.

## Methods

### Design and setting of the study

A cross-sectional study was conducted among inmates at the Tete City Prison, situated in the Tete Province of Mozambique, which is in Sub-Saharan Africa. The province is positioned in the central region of the country, sharing borders with Zambia to the northwest and Zimbabwe to the southeast. According to the 2017 Annual Report of the National Statistics Institute, the total population of the province is 2,764,169 inhabitants. The city of Tete, serving as the provincial capital, home to approximately 170,000 people. The prison under investigation is one of the main penitentiaries in the city [15]. The data were collected between March and April 2023.

### Population, sample size, and sampling techniques

The study population consisted of 1055 inmates of both sexes. Initially, a sample size of 282 participants was calculated using a mathematical formula for finite populations [16], considering a 50% expected response rate, a 95% confidence level (z-value = 1.96), and a margin of error of 0.05 [17]. These calculations were conducted using the Epi Info software [18]. To account for potential non-responses or exclusions, an additional 3% was added, resulting in a final sample size of 290 inmates.

To determine the sample size, we used the adjusted Cochran formula for finite populations:

$$\:n=\frac{p(1-p){Z}^{2}N}{{\text{d}}^{2}\left(N-1\right)+{Z}^{2}p(1-p)}$$

Given the population size (*N* = 1,055), a 95% confidence level (Z = 1.96), maximum variability (*p* = 0.5), and a 5% margin of error (d = 0.05), the sample size was calculated to be 290 participants. This approach is consistent with the guidelines for sample size determination outlined by Charan and Biswas [17].

The sample was selected using a probabilistic sampling technique, ensuring that each inmate had an equal chance of being included. First, a master list of all inmates was generated and numbered, from which participants in the study were randomly selected. Exclusion criteria were applied to inmates who did not comply with the required fasting period, refused to sign the informed consent form, were involved in activities that made their participation unfeasible, or were absent from the facility during the data collection period. Inmates excluded from the study were randomly replaced to maintain the sample size.

### Variables and measurements

All consenting participants were surveyed using an adapted questionnaire, developed based on previously published studies that employed the FINDRISK (Finnish Diabetes Risk Score) to identify risk factors for diabetes mellitus [19–22]. The same questionnaire was also adapted to assess the prevalence of hypertension among prisoners and its associated factors (See the supplementary file (S1). The questionnaire included questions about: (i) sociodemographic characteristics (age, sex, height, weight, and fasting blood glucose levels); (ii) risk factors for diabetes (self-reported diagnosis and treatment of hypertension, family history of diabetes, smoking status, and family history of hypertension); (iii) physical activity levels (frequency and average duration of exercise sessions per week); and (iv) dietary characteristics (number of daily meals, consumption of sugar, fats/oils, salt, and frequency of fruit and vegetable consumption).

All participants underwent a physical examination, which included anthropometric measurements (height and weight). Height (in meters) was measured with 0.1 mm precision using a wooden platform and a height ruler, without shoes, feet together, arms at the side of the body, and in an upright position on the wooden platform. Weight (in kg) was measured with 0.5 kg precision using a scale with the inmate wearing only light clothing. Body Mass Index (BMI) was calculated as weight (in kg) divided by height (in m²) [23]. Fasting capillary glucose was measured using a SensoLite Nova brand electronic glucometer using disposable strips. For the blood sample collection, prisoners were instructed to maintain a fasting period of at least 8 h before the blood sample tests.

Participants were asked if they had been diagnosed with diabetes by a doctor or health professional previously. This condition was measured dichotomously (yes or no). Those without a previous diagnosis underwent evaluation and were classified according to the criteria defined by the International Expert Committee. Individuals with fasting plasma glucose (FPG) levels ≥ 126 mg/dL (7.0 mmol/L) were considered diabetic, while those with FPG between 100 and 125 mg/dL (5.6 to 6.9 mmol/L) were classified as having prediabetes [24]. Individuals self-reporting as hypertensive or undergoing treatment for hypertension were considered hypertensive. Obesity was defined as a BMI of ≥ 30 kg/m², while overweight was defined as a BMI of 25.0 to 29.9 kg/m², and underweight was defined as a BMI below 18.5 kg/m². A sedentary lifestyle was defined as the absence of any physical activity [23].

### Data analysis

The medians and interquartile ranges (IQRs) were reported for quantitative variables such as age and BMI, while qualitative variables were presented as frequencies and percentages. A binomial test was used to calculate prevalence along with 95% confidence intervals (CIs). Group comparisons were conducted using Fisher’s exact test. An initial bivariable analysis was conducted using crude odds ratios (COR) to analyze factors associated with DM and hypertension. For the multivariable models (adjusted odds ratios, AOR), multicollinearity was assessed through correlation analysis and confirmed using the generalized variance inflation factor (GVIF). Initially, all variables were included in the multivariable analysis for a comprehensive exploration. Subsequently, the reduced multivariable model included variables with at least one category showing a p-value < 0.25 [25, 26]. To select the best-fitting model, the Akaike Information Criterion (AIC) was used, balancing model fit with complexity and penalizing models with more parameters. Lower AIC values indicate a better balance between fit and complexity [27]. Results were considered statistically significant for p-values < 0.05. Data analysis was performed using R software, version 4.3.2, and RStudio, version 2023.12.0 + 369.

## Results

### Characteristics of the study participants

The median age of the 290 participants was 29 years (IQR: 26–40). Regarding anthropometric measurements, weight and height were 61.2 kg (IQR: 59–69) and 1.76 m (IQR: 1.70–1.79), respectively. The median glycemic level recorded was 5.70 mmol/L (IQR: 4.6–6.08), and the calculated median BMI was 20.83 (IQR: 18.27–24.22).

The prevalence of prediabetes was 42.41% (95% CI: 36.66; 48.33; 123/290), and DM was diagnosed in 10.34% of participants (95% CI: 7.09; 14.44; 30/290). The prevalence of hypertension was 36.90% (95% CI: 31.33; 42.73; 107/290). The prevalence of obesity was 1.38% (95% CI: 0.38; 3.49), while 20% were classified as overweight (95% CI: 15.55; 25.07). A significant proportion, 37.93%, were below the ideal weight (95% CI: 32.32; 43.79).

According to Table 1, most participants were aged < 30 years (61.7%). Within this group, 8.9% were DM and 38.0% hypertensive, without statistically significant difference (*p* = 0.430). Regarding sex, the majority were male (77.6%), with a significant difference in the prevalence of hypertension (*p* < 0.001). Among smokers (61.0%), 8.5% were diabetic and 33.3% hypertensive, without a statistically significant difference for both conditions (*p* = 0.134).

**Table 1:** Characteristics of participants in relation to DM and hypertension among inmates in the City of tete, Mozambique

		Outcomes					
Variables		DM			Hypertension		
	Total (*N* = 290)	No (*N* = 260)	Yes (*N* = 30)		No (*N* = 183)	Yes (*N* = 107)	
		n (%)	n (%)	*p*-value	n (%)	n (%)	*p*-value
Age (years)							
< 30	179 (61.7%)	163 (91.1)	16 (8.9)		111 (62.0)	68 (38.0)	
30–40	51 (17.6%)	45 (88.2)	6 (11.8)	0.518	30 (58.8)	21 (41.2)	0.430
>41	60 (20.7%)	52 (86.7)	8 (13.3)		42 (70.0)	18 (30.0)	
Sex							
Male	225 (77.6%)	203 (90.2)	22 (9.8)		154 (68.4)	71 (31.6)	
Female	65 (22.4%)	57 (87.7)	8 (12.3)	0.644	29 (44.6)	36 (55.4)	< 0.001*
Nutritional status							
Normal weight	118 (40.7%)	102 (86.4)	16 (13.6)		76 (64.4)	42 (35.6)	
Underweight	110 (37.9%)	100 (90.9)	10 (9.1)		73 (66.4)	37 (33.6)	
Overweight	58 (20.0%)	55 (94.8)	3 (5.2)	0.185	32 (55.2)	26 (44.8)	0.470
Obesity	4 (1.4%)	3 (75.0)	1 (25.0)		2 (50.0)	2 (50.0)	
Physical Activities							
No	167 (57.6%)	149 (89.2)	18 (10.8)		105 (62.9)	62 (37.1)	
Yes	123 (42.4%)	111 (90.2)	12 (9.8)	0.847	78 (63.4)	45 (36.6)	1.000
Frequency of Physical Activity (min/day)							
≤ 20	79 (27.2%)	70 (88.6)	9 (11.4)		49 (62.0)	30 (38.0)	
>20	44 (15.2%)	41 (93.2)	3 (6.8)	0.797	29 (65.0)	15 (34.1)	0.927
Sedentary	167 (57.6%)	149 (89.2)	18 (10.8)		105 (62.9)	62 (37.1)	
Smoking							
Yes	177 (61.0%)	162 (91.5)	15 (8.5)		118 (66.7)	59 (33.3)	
No	113 (39.0%)	98 (86.7)	15 (8.5)	0.236	65 (57.5)	48 (42.5)	0.134
Family History with HTN							
Yes	115 (39.7%)	100 (87.0)	15 (13.0)		76 (66.1)	39 (33.9)	
No	100 (34.5%)	95 (95.0)	5 (5.0)		60 (60.0)	40 (40.0)	0.655
Unknown	75 (25.9%)	65 (86.7)	10 (13.3)	0.080	47 (62.7)	28 (37.3)	
Family History with DM							
Yes	108 (37.2%)	81 (75.0)	27 (25.0)		66 (61.1)	42 (38.9)	
No	84 (29.0%)	82 (97.6))	2 (2.4)	< 0.001*	48 (57.1)	36 (42.9)	0.156
Unknown	98 (33.8%)	97 (99.0)	1 (1.0)		69 (70.4)	29 (29.6)	
Fruit Consumption							
Yes	206 (71.0%)	182 (88.3)	24 (11.7)		131 (63.6)	75 (36.4)	
No	84 (29.0%)	78 (92.9)	6 (7.1)	0.294	52 (61.9)	32 (38.1)	0.790
Vegetable Consumption							
1–2 times	90 (31.0%)	81 (90.0)	9 (10.0)		57 (63.3)	33 (36.7)	
3–4 times	154 (53.1%)	138 (89.6)	16 (10.4)	1.00	90 (58.4)	64 (41.6)	0.048*
>4 times	46 (15.9%)	41 (89.6)	5 (10.9)		36 (78.3)	10 (21.7)	
Salt Consumption							
< 1 Tablespoons	17 (5.9%)	16 (94.1)	1 (5.9)		8 (47.1)	9 (52.9)	
1 Tablespoon	227 (78.3%)	204 (89.9))	23 (10.1)	0.757	145 (63.9)	82 (36.1)	0.374
>1 Tablespoons	46 (15.9%)	40 (87.0)	6 (13.0)		30 (65.2)	16 (34.8)	
Sugar Consumption							
≤ 5 Tablespoons	104 (35.9%)	93 (89.4)	11 (10.6)		68 (65.4)	36 (34.6)	
>5 Tablespoons	186 (64.1%)	167 (89.8)	19 (10.2)	1.00	115 (61.8)	71 (38.2)	0.612

A family history of diabetes was reported more frequently among participants with DM (25.0%) compared to those without (2.4%), showing a statistically significant association (*p* < 0.001). Regarding sugar consumption, there was no statistically significant difference between the groups (*p* = 1.00), with 10.2% consuming more than 5 tablespoons daily and 10.6% consuming less or equal to 5 tablespoons. A significant difference was observed in the prevalence of hypertension concerning vegetable consumption (*p* = 0.048). Hypertension was more common among individuals who consumed vegetables 3 to 4 times a week (41.6%) compared to those with other consumption frequencies.

### Risk factors associated with diabetes mellitus and hypertension in inmates

Bivariable and multivariable logistic regression analyses were conducted to identify factors associated with DM and hypertension. For DM, bivariable analysis revealed a higher prevalence among individuals with a family history of the condition (COR: 13.7; 95% CI: 13.67; 59.37), as shown in Table 2. To assess potential multicollinearity among the explanatory variables, the GVIF was calculated, with values ranging from 1.11 to 1.44 and an overall mean of 1.33, indicating that multicollinearity was not a concern (see Table S1). In the multivariable analysis, a family history of DM remained a significant risk factor (AOR: 14.95; 95% CI: 3.32; 67.44).

**Table 2:** Unadjusted and adjusted logistic regression model of risk factors for DM among inmates in the City of tete, Mozambique

Variables	Bivariable model		Multivariable model		Multivariable model reduced	
	COR (CI 95%)	*P*-value	AOR (CI 95%)		AOR (CI 95%)	*P*-value
Age (Years)						
< 30	Ref		Ref		Ref	
30–40	1.36 (0.50; 3.67)	0.546	1.56 (0.48; 5.13)	0.462	1.48 (0.46; 4.77)	0.507
>41	1.57 (0.63; 3.87)	0.330	2.24 (0.74; 6.74)	0.152	2.25 (0.78; 6.49)	0.132
Sex						
Male	Ref		Ref			
Female	1.30 (0.55; 3.06)	0.556	1.09 (0.34; 3.46)	0.887		
Nutritional status						
Normal weight	Ref		Ref		Ref	
Underweight	0.64 (0.28; 1.47)	0.292	0.58 (0.22; 1.58)	0.287	0.55 (0.21; 1.43)	0.223
Overweight	0.35 (0.10; 1.25)	0.105	0.26 (0.06; 1.11)	0.070	0.26 (0.06; 1.07)	0.061
Obesity	2.13 (0.21; 21.7)	0.525	7.97 (0.41; 156.38)	0.171	5.36 (0.25; 113.10)	0.280
Physical Activity						
>20	Ref		Ref			
≤ 20	1.76 (0.45; 6.86)	0.439	2.26 (0.42; 12.17)	0.272	-----------------------	---------------
Sedentary	1.65 (0.46; 5.88)	0417	2.35 (0.51; 10.80)	0.344	-----------------------	---------------
Smoking						
No	Ref		Ref		Ref	
Yes	0.60 (0.28; 1.29)	0.194	0.53 (0.20; 1.35)	0.180	0.52 (0.22; 1.26)	0.148
Hypertension						
No	Ref		Ref			
Yes	0.99 (0.45; 2.17)	0.978	0.79 (0.28; 2.24)	0.664	-----------------------	---------------
Family History with HTN						
No	Ref		Ref		Ref	
Yes	2.85 (1.00; 8.15)	0.050	2.60 (0.77; 8.78)	0.123	2.37 (0.74; 7.57)	0.146
Unknown	2.92 (0.95; 8.95)	0.060	2.52 (0.67; 9.48)	0.171	2.32 (0.67; 8.06)	0.184
Family History with DM						
No	Ref		Ref		Ref	
Yes	13.7 (13.67; 59.37)	< 0.001*	17.2 (3.60; 81.99)	< 0.001**	14.95 (3.32; 67.44)	< 0.001**
Unknown	0.42 (0.04; 4.75)	0.485	0.25 (0.02; 3.35)	0.295	0.33 (0.03; 3.94)	0.384
Fruit Consumption						
Yes	Ref		Ref		Ref	
No	0.58 (0.23; 1.48)	0.258	0.46 (0.15; 1.41)	0.172	0.44 (0.15; 1.28)	0.130
Vegetable Consumption						
1–2 times	Ref		Ref			
3–4 times	1.04 (0.44; 2.47)	0.923	0.55 (0.19; 1.60)	0.274	-----------------------	---------------
>4 times	1.10 (0.35; 3.49)	0.875	0.45 (0.11; 1.83)	0.264	-----------------------	---------------
Salt Consumption						
< 1 Tablespoons	Ref		Ref			
1 Tablespoon	0.50 (0.19; 1.35)	0.173	1.27 (0.13; 12.67)	0.838	-----------------------	---------------
>1 Tablespoons	0.47 (0.15; 1.47)	0.195	2.12 (0.17; 25.87)	0.556	-----------------------	---------------
Sugar Consumption						
≤ 5 Tablespoons	Ref		Ref			
>5 Tablespoons	0.96 (0.44; 2.11)	0.923	1.33 (0.51; 3.45)	0.557	-----------------------	---------------
AIC			174.54		160.72	

Similarly, for hypertension, bivariable analysis indicated an increased prevalence among female inmates (COR: 2.69; 95% CI: 1.53; 4.73), as presented in Table 3. GVIF values ranged from 1.05 to 1.18, with an overall mean of 1.25, again suggesting no significant multicollinearity (see Table S2). After adjusting for confounding factors, female gender remained significantly associated with hypertension (AOR: 2.54; 95% CI: 1.43; 4.52).

**Table 3:** Unadjusted and adjusted logistic regression models of risk factors for hypertension among inmates in the City of tete, Mozambique

Variables	Bivariable model		Multivariable model		Multivariable model reduced	
	COR (CI 95%)	*P*-value	AOR (CI 95%)	*P*-value	AOR (CI 95%)	*P*-value
Age (Years)						
< 30	Ref		Ref			
30–40	1.14 (0.61; 2.15)	0.680	1.09 (0.55; 2.16)	0.797	----------------------	---------------
>41	0.70 (0.37; 1.31)	0.266	0.75 (0.38; 1.44)	0.383	----------------------	---------------
Sex						
Male	Ref		Ref		Ref	
Female	2.69 (1.53; 4.73)	< 0.001**	2.51 (1.38; 4.54)	0.002**	2.54 (1.43; 4.52)	0.001*
Nutritional status						
Normal weight	Ref		Ref			
Underweight	0.92 (0.53; 1.58)	0.756	0.89 (0.49; 1.59)	0.687	----------------------	---------------
Overweight	1.47 (0.78; 2.79)	0.238	1.39 (0.69; 2.77)	0.355	----------------------	---------------
Obesity	1.81 (0.25; 13.32)	0.560	1.83 (0.22; 15.34)	0.579	----------------------	---------------
Physical Activity						
>20	Ref		Ref			
≤ 20	1.18 (0.55; 2.56)	0.668	0.97 (0.42; 2.22)	0.945	----------------------	---------------
Sedentary	1.14 (0.57; 2.29)	0.710	1.00 (0.48; 2.09)	0.995	----------------------	---------------
Smoking						
No	Ref		Ref		Ref	
Yes	0.68 (0.42; 1.10)	0.116	0.72 (0.41; 1.19)	0.185	0.71 (0.43; 1.18)	0.188
Diabetes Mellitus						
No	Ref		Ref			
Yes	0.99 (0.45; 2.17)	0.978	0.99 (0.42; 2.31)	0.978	----------------------	---------------
Family History with HTN						
No	Ref		Ref			
Yes	0.77 (0.44; 1.34)	0.356	0.72 (0.40; 1.30)	0.273	----------------------	---------------
Unknown	0.89 (0.48; 1.65)	0.720	0.75 (0.38; 1.46)	0.397	----------------------	---------------
Fruit Consumption						
Yes	Ref		Ref			
No	1.07 (0.64; 1.82)	0.787	0.92 (0.52; 1.61)	0.760	----------------------	---------------
Vegetable Consumption						
1–2 times	Ref		Ref		Ref	
3–4 times	1.23 (0.72; 2.10)	0.452	1.43 (0.80; 1.61)	0.232	1.37 (0.79; 2.38)	0.267
>4 times	0.48 (0.21; 1.09)	0.080	0.62 (0.26; 1.49)	0.287	0.59 (0.25; 1.37)	0.222
Salt Consumption						
< 1 Tablespoons	Ref		Ref			
1 Tablespoon	1.99 (0.74; 5.35)	0.173	0.55 (0.20; 1.57)	0.266	----------------------	---------------
>1 Tablespoons	2.11 (0.68; 6.52)	0.195	0.63 (0.19; 2.12)	0.457	----------------------	---------------
Sugar Consumption						
≤ 5 Tablespoons	Ref		Ref			
>5 Tablespoons	1.17 (0.71; 1.92)	0.547	1.01 (0.59; 1.73)	0.978	----------------------	---------------
AIC			395.43		373.04	

## Discussion

We conducted this study to determine the prevalence of NCDs and their determinants among inmates in Tete, Mozambique, representing the first research conducted in a specific prison population in the country. Two health outcomes (hypertension and DM) were considered in the bivariable and multivariable models.

The overall prevalence of hypertension among inmates in Tete City was 36.90%, while the prevalence of pre-diabetes and DM was 42.41% and 10.34%, respectively. An obesity rate of 1.38% was observed, and overweight reached 20.0%. Regarding hypertension, a significant agreement was found in the results, with similar prevalences identified in various previously published studies, including a systematic review [14], as well as studies conducted in Cameroon [28] and in a prison in the state of Arizona, United States [29]. However, considerable variations were observed in other locations, such as India [30] and northern Ghana [31]. The findings of this specific population study did not differ substantially from the prevalence observed in South Africa, Limpopo Province, which was 38.2% [32]. Regarding the prevalence of pre-diabetes and DM, it is noteworthy that disparate results were identified in other regions, such as northern Ghana, where the rates of DM and pre-diabetes were relatively low [31], as well as in India [30]. Similar results were found in Cameroon [13]. Outside of sub-Saharan Africa, rates ranged from 2.5 to 5.3% [24, 25], including locations like Arizona, where the diabetes mellitus rate was 7.7% [29]. These discrepancies suggest the influence of regional and demographic factors on the prevalence of hypertension and DM in prison contexts, including considerations such as the age of the inmates [33]. People living in prisons experience increased rates of chronic NCDs when compared to the general population [34]. The increase in the prevalence of NCDs, such as hypertension and DM, can be attributed to a variety of factors, including individual, community, and structural aspects that precede incarceration, as well as the prison environment itself [35]. Individual factors encompass diet, physical activity levels, and substance use; community factors include adverse childhood experiences and exposure to trauma; and structural factors refer to limited access to healthcare, housing insecurity, and food insecurity. Furthermore, the prison environment contributes to the creation of risk factors that likely exacerbate health outcomes, resulting in acute and chronic stress due to dehumanizing conditions, exposure to violence, poor nutrition, and unequal access to physical activity and quality healthcare [35, 36]. In Sub-Saharan Africa, particularly, prisons are characterized by severe and potentially life-threatening conditions, including overcrowding, poor sanitation, and a lack of access to basic necessities such as sufficient food, security, healthcare, potable water, and heating [37, 38].

The prevalence of obesity and overweight in this study was 1.38% and 20.0%, respectively. This evidence contrasts with the divergent findings observed in previous studies, such as those conducted in Cameroon [13] and northern Ghana [31]. When expanding the analysis beyond African borders, significant disparities can be identified. In Arizona, for example, the overweight rate was significantly higher, reaching 36.8%, while the prevalence of obesity was 22.8% [29]. This variation suggests the influence of multifactorial factors, including cultural, socioeconomic, and environmental aspects that contribute to these different prevalences.

In this research, the adjusted logistic regression analysis revealed a significant association between being female and the prevalence of hypertension among inmates. This finding can be explained by the specific challenges faced by women in the prison environment, including sexual and reproductive health needs and inadequate nutrition, exacerbated by overcrowding and poor sanitation [35]. These factors are common in prisons across Sub-Saharan Africa, where access to quality healthcare is limited. Moreover, women encounter psychosocial stressors, such as gender-based violence and a lack of support for their basic needs, as well as the additional stress of either living with their children in prison or being separated from them, which profoundly impacts their emotional well-being. The combination of these factors, along with a frequently unfavorable health history, contributes to the higher prevalence of hypertension among female inmates compared to other groups [35, 38].

Regarding DM, after adjusting the model, family history of DM remained a significant risk factor associated with the condition, as observed in studies conducted in Ethiopia [39]. Family history of diabetes, a non-modifiable risk factor [40], is widely recognized as an important risk factor for the incidence of DM in offspring [41, 42]. The risk of developing DM increases approximately two to four times when one or both close relatives have the condition [43]. This elevated risk may be partly mediated by genetic components and shared environmental factors among family members, although the specific factors driving this increased risk are not yet fully understood [44]. Lifestyle-related and anthropometric risk factors, such as BMI, waist circumference, and physical inactivity, are also important determinants for DM [45]. The aggregation of these traits within families may account for part of the excess risk attributable to family history [46].

Given the considerable prevalence of hypertension, DM, and other chronic health conditions and their associated risk factors identified among inmates, it is crucial to implement effective preventive strategies that consider the adverse conditions often present in the African context. These conditions include overcrowding, inadequate sanitation, and a lack of access to healthcare, nutrition, security, and potable water. To address these challenges, a comprehensive approach is necessary, ranging from careful screening of individuals with multiple risk factors to dietary improvements, policies to reduce excessive sugar consumption, and the promotion of regular physical activity. In penitentiary settings, inmates have less control over their risk factors compared to those in the community, with limited choices regarding diet and exercise. Many risk factors are socially determined rather than mere lifestyle choices [14]. Therefore, it is vital to implement policies that acknowledge these constraints and expand health teams within prisons. These teams should be trained to identify and manage chronic health conditions while addressing the specific needs of different groups, such as women, who face additional challenges related to reproductive health and psychosocial stress. These considerations underscore the need for integrated and comprehensive approaches to effectively tackle chronic diseases in prisons, as health promotion activities are essential for maintaining inmate health [47].

This study has certain limitations. Its cross-sectional design prevents establishing temporality between inmates and risk factors for chronic conditions. Although a random sampling method was used in Tete prisons, findings cannot be generalized to all Mozambican inmates due to the study’s limited scope. Additionally, diabetes diagnosis relied on fasting capillary glucose without oral glucose tolerance or glycated hemoglobin tests. Despite these limitations, this is the first study of its kind in Mozambique, providing valuable insights into NCDs prevalence and risk factors among inmates. Its comprehensive approach offers a deeper understanding of incarcerated individuals’ health challenges. Future studies incorporating direct blood pressure measurements and a broader array of variables, ideally through multicentric designs, would enhance hypertension assessment and deepen our understanding of NCDs in incarcerated populations. This study lays important groundwork for future research and the development of targeted prison health policies.

## Conclusion

Our findings indicate a high prevalence of diabetes and hypertension among prisoners, particularly hypertension among women and diabetes among those with a family history. These results underscore the importance of specific and targeted approaches to the health of this vulnerable population, including regular screenings, health education, improved dietary practices, and continuous care. Such strategies are essential not only for early identification but also for the effective management of these chronic conditions in prison settings.

## Supplementary information

Supplementary Material 1.

Supplementary Material 2.

## Data Availability

The datasets used and/or analyzed during the current study are available from the corresponding author upon reasonable request.
