# Supplementary material for: Prevalence and determinants of chronic non-communicable diseases among prison inmates in the city of Tete, Mozambique: a cross-sectional study
Source: BMC Public Health. 2025 Sep 24;25:3110. doi: 10.1186/s12889-025-24387-4 (PMC12462332; doi:10.1186/s12889-025-24387-4)
Supplement: Supplementary file 1 — Supplementary Material 1. [file 12889_2025_24387_MOESM1_ESM.docx]

**Supplementary file (S1)**

**Questionnaire on Factors Associated with the Prevalence of Non-Communicable Chronic Diseases (NCDs) among Prison Inmates in the City of Tete, Mozambique**

- 1. **SOCIO-DEMOGRAPHIC DATA**
  2. Name (initials only): [ ]
  3. Age: [____]
  4. Gender: [___]
  5. Height (cm): [______]
  6. Weight (kg): [______]
  7. Fasting glucose (mg/dL): [______]
  8. Body Mass Index (BMI) (calculated from height and weight): [_____]
  9. **ASSOCIATED RISK FACTORS**

**Hypertension**

1. Have you been diagnosed with hypertension by a healthcare professional?

[___] Yes [___] No [___] I don’t know

2. Are you taking any medication to control hypertension?

[___] Yes [___] No

3. Do you have family members with hypertension?

[___] Yes [___] No [___] I don’t know

4. What is the relationship with the family member(s) with hypertension?

[___] Father [___] Mother [___] both [___] Others

**Diabetes Mellitus (DM)**

1. Have you been diagnosed with diabetes mellitus by a healthcare professional?

[___] Yes [___] No [___] I don’t know

2. Do you have family members with diabetes mellitus?

[___] Yes [___] No [___] I don’t know

3. What is the relationship with the family member(s) with diabetes?

[___] Father [___] Mother [___] both [___] Others

**Other Habits**

1. Do you smoke or have you smoked in the past?

[___] Yes [___] No

- 1. **PHYSICAL ACTIVITY LEVELS**

3.1. How often do you engage in physical exercise?

[___] None [___] 1 to 2 days per week [___] 3 to 4 days per week

[___] 5 to 6 days per week [___] Every day

- 1. **DIETARY HABITS**

4.1. How many meals do you have per day?

[ ] 1 to 2 [ ] 3 to 4 [ ] 5 to 6

4.2. Sugar consumption:

[___] High (more than 5 teaspoons per day)

[___] Medium (up to 5 teaspoons per day)

[___] Low (less than 5 teaspoons per day)

[___] None

4.3. Fat and oil consumption

[___] High (more than 1 tablespoon per day)

[___] Medium (1 tablespoon per day)

[___] Low (less than 1 tablespoon per day)

[___] None

4.4. Salt consumption:

[___] High (more than 5g per day)

[___] Medium (5g per day)

[___] Low (less than 5g per day)

[___] None

4.5. Fruit consumption:

[___] Yes [___] No [___] Sometimes

If "yes", how many times per week?

[___] 1 to 2 [___] 3 to 4 [___] 5 to 6 [___] 7 days per week

4.6. Vegetable and legume consumption:

[___] 1 to 2 times per week [___] 3 to 4 times per week

[___] 5 to 6 times per week [___] Every day [___] None
