# Supplementary material for: Prevalence and determinants of chronic non-communicable diseases among prison inmates in the city of Tete, Mozambique: a cross-sectional study
Source: BMC Public Health. 2025 Sep 24;25:3110. doi: 10.1186/s12889-025-24387-4 (PMC12462332; doi:10.1186/s12889-025-24387-4)
Supplement: Supplementary file 2 — Supplementary Material 2. [file 12889_2025_24387_MOESM2_ESM.docx]

**Supplementary file (S2)**

**S2.** Generalized Variance Inflation Factor (GVIF) and Adjusted GVIF for Explanatory Variables in the Model for Hypertension

| **Variable** | **GVIF** | **Df** | **GVIF^(1/(2*Df))** |
| --- | --- | --- | --- |
| Age | 1.08 | 2 | 1.02 |
| BMI | 1.18 | 3 | 1.03 |
| Sex | 1.06 | 1 | 1.03 |
| Time in physical activity | 1.08 | 2 | 1.02 |
| Smoking status | 1.08 | 1 | 1.04 |
| Diabetes | 1.05 | 1 | 1.03 |
| Family history of hypertension | 1.12 | 2 | 1.03 |
| Consumption of fruits | 1.07 | 1 | 1.03 |
| Consumption of vegetables | 1.18 | 2 | 1.04 |
| Tablespoons of salt | 1.12 | 2 | 1.03 |
| Tablespoons of sugar | 1.06 | 1 | 1.03 |

Note: GVIF = Generalized Variance Inflation Factor; Df = Degrees of Freedom; GVIF^(1/(2*Df)) = Square root of GVIF divided by twice the degrees of freedom.

**S2:** Generalized Variance Inflation Factor (GVIF) and Adjusted GVIF for Explanatory Variables in the Model for Diabetes Mellitus

| **Variable** | **GVIF** | **Df** | **GVIF^(1/(2*Df))** |
| --- | --- | --- | --- |
| Age (categorized) | 1.22 | 2 | 1.05 |
| Sex | 1.34 | 1 | 1.16 |
| BMI (categorized) | 1.44 | 3 | 1.06 |
| Time of physical activity | 1.26 | 2 | 1.06 |
| Smoking | 1.17 | 1 | 1.08 |
| Hypertension (categorized) | 1.32 | 1 | 1.15 |
| Family history of hypertension | 1.23 | 2 | 1.05 |
| Family history of diabetes | 1.29 | 2 | 1.06 |
| Consumption of fruits | 1.15 | 1 | 1.07 |
| Consumption of vegetables | 1.26 | 2 | 1.06 |
| Tablespoons of salt | 1.27 | 2 | 1.06 |
| Tablespoons of sugar | 1.11 | 1 | 1.05 |

GVIF = Generalized Variance Inflation Factor; Df = Degrees of Freedom; GVIF^(1/(2*Df)) = Square root of GVIF divided by twice the degrees of freedom.
